# Supplementary material for: Biomimetic KcsA channels with ultra-selective K+ transport for monovalent ion sieving
Source: Nat Commun. 2022 Mar 31;13:1701. doi: 10.1038/s41467-022-29382-6 (PMC8971412; doi:10.1038/s41467-022-29382-6)
Supplement: Supplementary file 1 — Supplementary Information [file 41467_2022_29382_MOESM1_ESM.pdf]

Supplementary Information for

Biomimetic KcsA channels with ultra-selective K^+ transport for monovalent ion sieving

Weiwen Xin[1,2,4], Jingru Fu[3,4], Yongchao Qian[1], Lin Fu[1,2], Xiang-Yu Kong[1], Teng Ben[3,*], Lei Jiang[1,2], Liping Wen[1,2,*]

Affiliations:

[1]*Key Laboratory of Bio-inspired Materials and Interfacial Science, Technical Institute of Physics and Chemistry, Chinese Academy of Sciences, Beijing 100190, P. R. China.*

[2]*School of Future Technology, University of Chinese Academy of Sciences, Beijing 100049, P. R. China.*

[3]*Department of Chemistry, Jilin University, Changchun 130012, P. R. China.*

[4]*These authors contributed equally.*

**Correspondence and requests for materials should be addressed to L.W. (email: wen@mail.ipc.ac.cn) or to T.B. (tben@jlu.edu.cn).*

Table of content

1. Supplementary Materials and Methods

1.1. Chemicals.

1.2. Synthesis of compounds.

1.3. Single nanochannel fabrication.

1.4. Activated surface of single nanochannel.

1.5. CO_2 adsorption isotherm measurement.

1.6. Powder X-ray diffraction (PXRD).

1.7. Scanning electron microscopy (SEM).

1.8. X-ray photoelectron spectroscopy (XPS) measurements.

1.9. Temperature-dependent conductivity.

1.10. Ion concentration measurements for ternary ion permeation.

1.11. Rectification effect.

1.12. Ion sieving of the mixing solutions.

2. Supplementary Figures (1–14)

1. Structural details of the CPOS material.
2. Route of the preparation for the biomimetic K^+ ion channel.
3. SEM images of the base and tip of nanocone.
4. TGA plot of the CPOS material.
5. The experimental set-up.
6. The $I–V$ characteristics of the pristine channel in 0.1 M and 0.01 M monovalent ion solutions at a bias voltage from −1 to 0 V.
7. The normalization flux of different salt solutions.
8. High-resolution XPS spectra of the pristine CPOS samples.
9. FTIR spectra of the CPOS material at low wavenumber.
10. Schematic of ion radius (R_{ion}) and the first hydration shell radius (R_{min}).
11. The molecular snapshot of ions in the center of the screwing cavity.
12. RDFs of water molecules around anions sitting in bulk water.

13. Ionic current under a constant bias.
14. Ion selectivity in the quinary solutions.

3. Supplementary Tables (1–3)

1. Ion-dependent parameters including the ionic radius, hydration ionic radius, number of the first hydration shell, and hydration energy.
2. Monovalent ion selectivity based on various types of the artificial channels and membranes.
3. Experimental details of the zero-current voltage (E_m), E_{redox}, and real E_m values which could be calculated according the equation: real E_m=E_m-E_{redox}.

4. Supplementary References (1–21)

1. Supplementary Materials and Methods

1.1. Chemicals.

The monomers 4,4′,4″,4‴-methanetetrabenzenesulfonic acid (H_4TBS), terephtalimidamide hydrochloride (DAB·HCl) were synthesized according the previously published literature[1], as shown in Section **1.2**. Lithium chloride (LiCl), sodium chloride (NaCl), potassium chloride (KCl), magnesium chloride ($MgCl_2$), and calcium chloride ($CaCl_2$) were purchased from J&K Beijing Co., Ltd. 1-ethyl-3-(3-dimethyllaminopropyl) carbodiimide (EDC), N-hydroxysuccinimide (NHSS), 14% sodium hypochlorite (NaClO), ethanediamine ($C_2H_8N_2$), potassium iodide (KI), and tetrahydrofuran (THF) were purchased from Sigma-Aldrich. The polyimide (PI) membranes employed in this work are based on an UMAT experiment, performed at the beam line X0 at the GSI Helmholtzzentrum für Schwerionenforschung, Darmstadt (Germany) in the frame of FAIR Phase 0. All chemicals were used without further purification. All the experimental solutions were prepared using degassed Milli-Q water (18.2 MΩ cm^{-1}).

1.2. Synthesis of compounds.

Synthesis of tetraphenylmethane.

24.8 g triphenylmethanol (0.1 mol) was immersed in acetic acid (268 ml) to form a mixture. Subsequently, 13.7 ml phenylamine (0.15 mol) and 50 ml concentrated hydrochloric acid were added to the as-prepared mixture in a one-neck round-bottomed flask equipped with a condenser. The reaction solutions were heated at 125°C at this temperature under reflux for 2 days. The hot brown-yellow solution was collected and poured into 1200 ml saturated NaOH solution, resulting into the precipitation of white solid. The solid was collected by filtration, then washed with water until the solution became neutral. After drying, the obtained solid was immersed in a mixture of 714 ml ethanol and 46 ml concentrated sulfuric acid. 28 ml isoamyl nitrite was then added dropwise in the solution, and left to stir at −10°C for 50 min. After returning to room temperature, 80 ml hypophosphorous acid was added in the mixture, and then the reaction solution was heated at 90°C under reflux for 8 h and the obtained precipitate was isolated by filtration with water and ethyl alcohol. The waterish product was dried in drying oven overnight, giving 20.4 g tetraphenylmethane (yield: 64%).

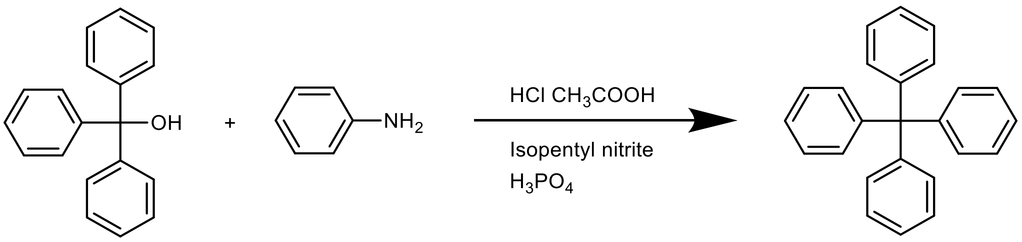

Synthesis of 4,4',4'',4'''-methane tetrabenzenesulfonate (TBS).

2.56 g tetraphenylmethane (8.0 mmol) was dispersed in 120 ml dichloromethane (CH_2Cl_2) and left to stir in ice-water bath. A mixture of 8.5 ml $ClSO_3H$ (128 mmol) and 80 ml CH_2Cl_2 was added dropwise in the solution until the reaction was finished, which was monitored using nuclear magnetic resonance. After standing for 5 min, the solid was collected by filtration, then washed with CH_2Cl_2. The treated solid recrystallized in a mixture of methanol and acetonitrile. The product was collected by vacuum filtration, giving 3.4 g 4,4′,4″,4‴-methane tetrabenzenesulfonate as a powder (yield: 50%).

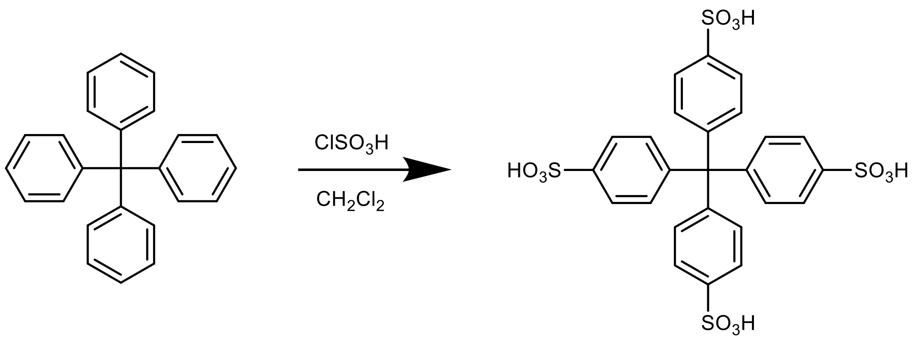

Synthesis of 1,4-diamidiniumbenzene dihydrochloride.

Under the protection of argon, 2.56 g terephthalonitrile was immersed in 30 ml absolute ethanol. The mixture was cooled to −15°C accompanied by HCl gas for 4 h. After returning to room temperature, the mixture was left to stir for 13 h. Subsequently, dry HCl gas was again turned on for 2 h, and the mixture was left to stir for 21 h at room temperature. After that, the solvent was removed by vacuum rotary evaporation. The white intermediate product was dried by vacuum at room temperature for 4 h, which was then immersed in 25 ml absolute ethanol and cooled to −15°C accompanied by dry NH_3 gas. After returning to room temperature, the mixture was left to stir for 63 h and the solvent was removed by vacuum rotary evaporation. The white crude product was collected by vacuum drying at room temperature for 4 h, which recrystallized in a mixture of water and ethanol, giving 2.8 g 1,4-diamidiniumbenzene dihydrochloride as a white crystalline powder (yield: 60%).

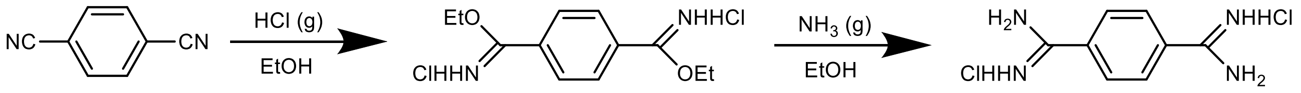

1.3. Single nanochannel fabrication.

A single conical nanochannel embedded in the PI membrane was prepared by the well-developed ion track-etching technique[2]. In detail, prior to the track etching, the two faces of the membrane were respectively activated by the ultraviolet light for 60 min. One side of the membrane was in contact with the etchant (14% NaClO), and the other side was in contact with the stopping solution (1.0 M KI). The etching was carried out at 60°C in a warm-box, and a constant voltage of 1.0 V was applied to monitor the process. The etching process was stopped at a desired current value corresponding to a certain tip diameter. When the ion track-etching was finished, the process remained continuing for 30 min. For all samples, the diameters of the bases were approximately 750 nm, and the diameters of the tips were approximately 75 nm, which could be observed using the SEM images.

1.4. Activated surface of single nanochannel.

To prepare the activated surface of the single nanochannel, we first immersed the PI membrane with a single conical nanochannel in an aqueous solution of EDC (15.0 mg ml^{-1}) and NHSS (3.0 mg ml^{-1}) for 1 hour. Then, we immersed the treated membrane in an aqueous solution of 50 mM $C_2H_8N_2$ at room temperature for 12 hours without any light to accomplish the amino surface coupling process. In order to achieve a complete modification for the whole channel, we adopted a bottom-up approach, that is from the tip to the base, to activate the surface of the single nanochannel. The tip of the channel was in contact with EDC/NHSS solutions, and the membrane was placed on the surface of the solutions. Finally, we cleaned the as-prepared membrane with Milli-Q water.

1.5. CO_2 adsorption isotherm measurement.

The CO_2 sorption experiments at 273 K up to 1 bar were collected using a Micromeritics ASAP 2020 surface area and pore size analyzer. Before the sorption analysis, the samples were evacuated at 150°C for overnight using a turbo molecular vacuum pump. The micropore surface area was calculated from the CO_2 adsorption data by the Dubinin-Astakhov method. The pore size distributions were calculated from the CO_2 adsorption isotherms using the non-local density functional theory (NLDFT) method. Ultrahigh purity grade CO_2 (99.999%) was used for the adsorption measurements. Helium (99.999%) was used to measure the free space. Gas isotherms at 273 K were carried out in an ice-water bath to keep the temperatures stable.

1.6. Powder X-ray diffraction (PXRD).

Powder X-ray diffraction (PXRD) measurements were accomplished with a PANalytical B.V. Empyrean powder diffractometer using Cu-Kα radiation at 40 kV and 40 mA over a range of $2\theta = 4.0$ to 40.0° with a step size of 0.02° and 2 second per step.

1.7. Scanning electron microscopy (SEM).

Field-emission SEM (Hitachi S-4800 scanning electron microscope with an accelerating voltage of 10 kV coupled with second electron imaging was performed to observed the structural details of the nanochannel. In order to obtain the high-quality image information, the Au nanolayer was deposited onto the surface of the nanochannel by ion sputtering with an Au target (99.999%) using an ion sputtering system (SBC-12, KYKY Technology Development Ltd.) in a vacuum (>4.0 MPa) for 60 seconds. Since the tip is too small to be found in such a large membrane, we used a porous membrane (10^7 nanochannels cm^{-2}) by appropriate etching for SEM observation to reflect the microscopic morphology of the tip region.

1.8. X-ray photoelectron spectroscopy (XPS) measurements.

XPS spectra were recorded in an ultrahigh-vacuum system with a base pressure of less than 5×10^{-10} mbar using an Al Ka source (ESCALAB 250Xi) and a power of 300 W. In order to confirm the strong interactions between the CPOS and metal ions, these samples were filled into the porous conical PI nanochannels (pore density: 10^7 nanochannels cm^{-2}) using the *in situ* growth strategy, which underwent a $I-V$ scanning in different salt solutions (LiCl, NaCl, and KCl). Subsequently, the porous membrane was washed for several times with MilliQ water to remove possible residual ions from the surface of the CPOS crystal. Dry membrane was investigated using XPS to reflect the binding effect of the CPOS and metal ions. The pristine CPOS samples were also measured as the contrast experiments.

1.9. Temperature-dependent conductivity.

At different temperatures, the $I-V$ curves were recorded by a Keithley 6487 picoammeter. The activation energy was therefore calculated from the conductivities as a function of temperature using the simplified Arrhenius equation (Supplementary Equation 1):

$$ln\,G = -\frac{E_a}{R}\frac{1}{T} + ln\,G_0 \qquad \text{(Supplementary Equation 1)}$$

where G is the conductivity, G_0 is a constant, E_a is the activation energy, R is the gas constant, and T is the absolute temperature. The log of the conductivity was then plotted as a function of the temperature. Thus, the E_a value could be calculated according to the slope of the fitting curves.

1.10. Ion concentration measurements for ternary ion permeation.

The metal ionic concentrations (permeation experiments) were carried out by a Keithley 6487 picoammeter with the applied –1 V voltage for 24 hours whereby the feed solutions were a mixture of the same concentration of LiCl, NaCl, and KCl, and the other chamber was filled with the deionized water as the permeation solution using the commercial Pt electrodes. At the end of the experiments, the metal ion concentrations in the permeation solution were affirmed employing the inductively coupled plasma optical emission spectrometry.

1.11. Rectification effect.

The ion transport property of a single conical nanochannel with and without the negatively charged CPOS can be characterized by the *I–V* curves. According to the previously report[3], the shape of the internal potential in a conical nanochannel is asymmetric. For a negatively charged conical nanochannel, only cations (such as K^+, Na^+, and Li^+ ions) of the electrolyte can translocate through the nanochannel. At positive voltage, when cations translocate from the base to the tip, the translocation becomes more and more difficult due to the narrowing of nanochannel. On the contrary, at negative voltage, when cations translocate from the tip to the base, the translocation becomes easier and easier due to the broadening of nanochannel. It was seen that the ionic current value at +1 V is much lower than that at –1 V. Therefore, to enhance the ion flux, the ion transport behavior and permeation experiments were conducted at –1 V bias.

1.12. Ion sieving of the mixing solutions.

The biomimetic nanochannel membrane was clamped between two PTFE compartments, of which one cell, facing the base of the conical nanochannel, was filled with a mixture of 100 mM LiCl, NaCl, KCl, RbCl, and CsCl as the feed solutions (10 ml), while the other cell was filled with MilliQ water as the permeation solution. The quinary ion selectivity measurements were conducted by applying a constant

potential of –1 V versus Pt electrodes using a Keithley 6487 picoammeter for 24 hours. Once these measurements completed, the permeation solutions were collected and then measured using the inductively coupled plasma mass spectrometry for further information on metal ion concentration.

2. Supplementary Figures

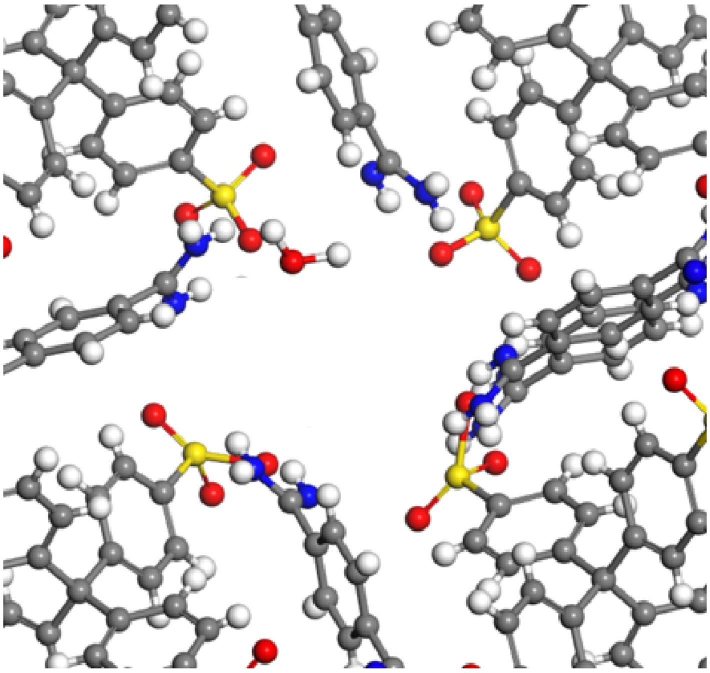

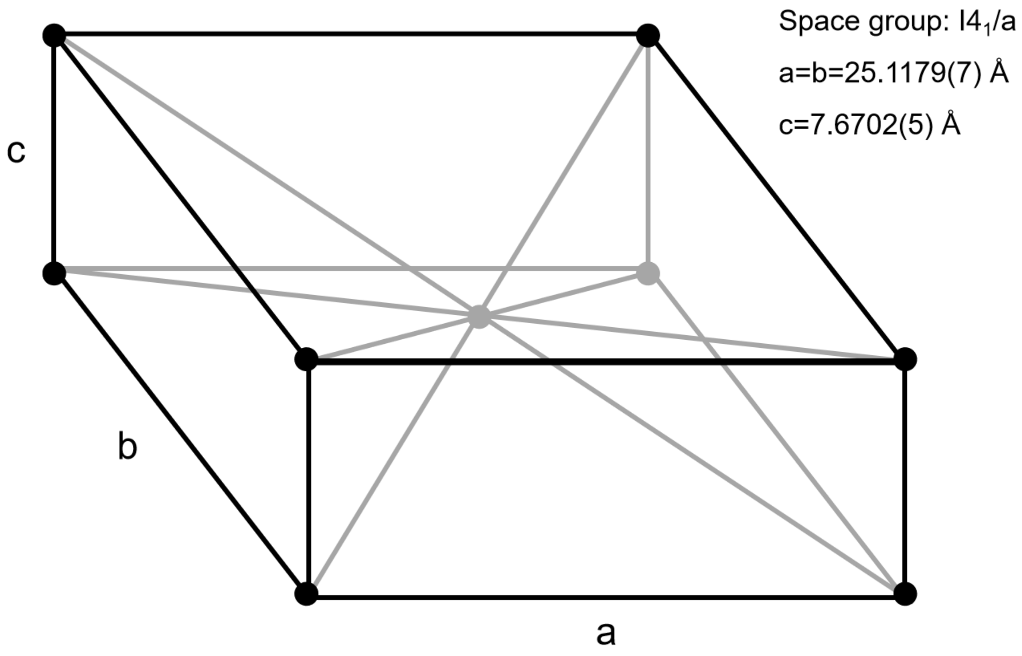

Supplementary Figure 1. Structural details of the CPOS material. Crystallizing occurs in the tetragonal $I4_1/a$ space group as illustrated ($a = b = 25.1179(7)$, $c = 7.6702(5)$; $\alpha = \beta = \gamma = 90°$), which contains a quarter of TBS and half of DAB in the asymmetric unit. A 4-fold rotoinversion axis crosses the central carbon, which orients four sulphonate groups (SO_3^-) along the vertices of a tetrahedron.

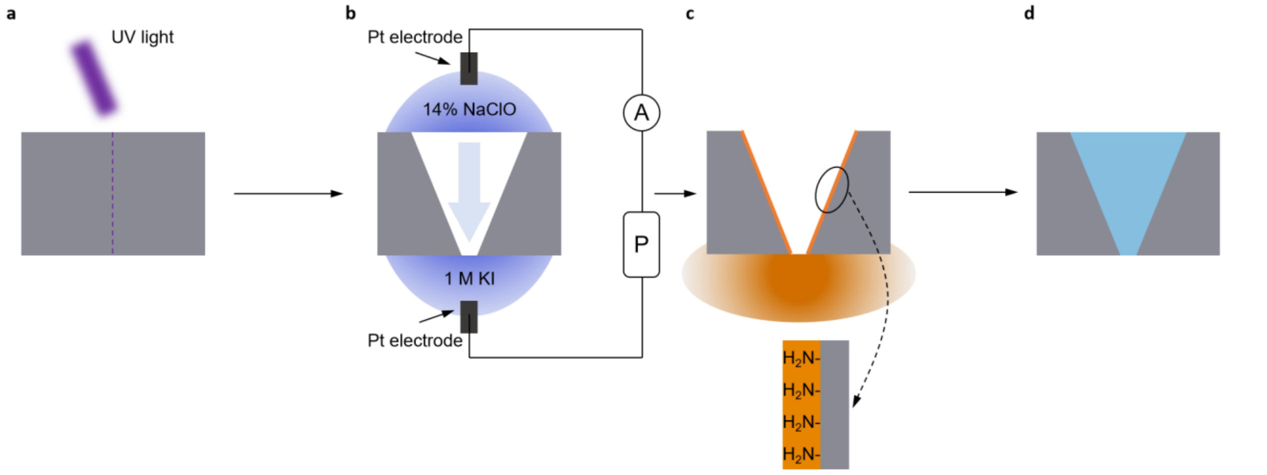

Supplementary Figure 2. Route of the preparation for the biomimetic K^+ ion channel. The process was divided into four steps including 1. irradiation of the PI membrane using UV light; 2. ion-track etching using NaClO solutions under an applied bias to obtain the single nanocone; 3. activating the inner surface to form an amino wall; 4. *in situ* growth of the CPOS material from the tip to the base. In detail, (a) The two faces of the polyimide (PI) polymer membrane were first irradiated with an ultraviolet (UV) light for one hour. (b) The single conical nanochannel was conducted using the etching technique at 333 K under 1 V bias. The PI membrane was clamped between two PTFE compartments, of which one cell, facing the base of the conical nanochannel, was filled with 14% NaClO solution as the etching solutions, while the other cell was filled with 1 M KI solution in order to neutralize the etchant as soon as the channel opened. (c) The carboxyl group surface was activated by soaking in EDC/NHSS aqueous solution for one hour at room temperature without light. Once finished, the samples were further treated with 50 mM ethanediamine overnight, resulting in the amino groups onto the surface of the nanocone. (d) The PI membrane was immersed into TBS solutions, and then the DAB solutions were added in a static state overnight, yielding *in situ* growth of the crystals from the tip to the base. Note that the tip of the channel was in contact with TBS/DAB solutions. Resultant membrane was washed for several times using H_2O/THF solutions. It should be pointed out that once the growth direction is reversed, the crystals in the nanochannel cannot be completely filled especially in the tip region, leading to a vacancy that could produce a deviation of ion transport.

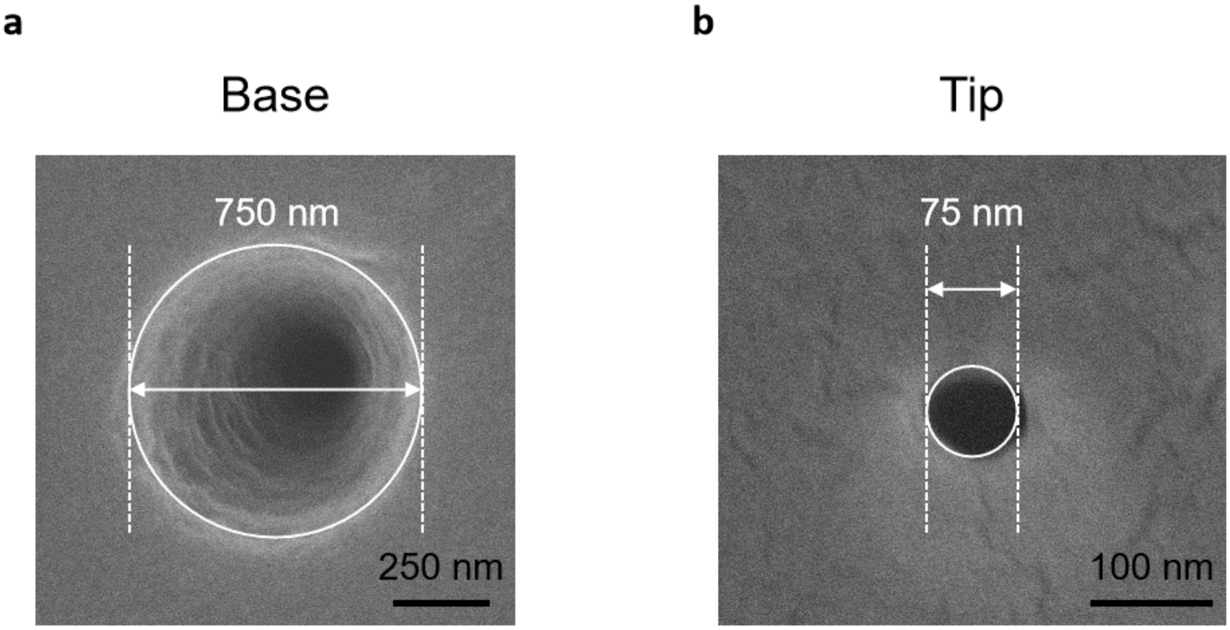

Supplementary Figure 3. SEM images of the base and the tip of the nanocone. As a typical example of showing the dimension of the conical channel, we can calculate the diameter of the base to be 750 nm (a). The diameter of the tip can be found to be ~75 nm (b) in a porous membrane (10^7 nanochannels cm^{-2}), which is in good agreement with the calculated result by the equation (Supplementary Equation 2):

$$d_{tip} = \frac{4Il}{\pi \kappa_c U D} \quad \text{(Supplementary Equation 2)}$$

where κ_c is the specific conductivity in 1 M KCl solution at 298 K, that is, 0.11173 Ω^{-1} cm^{-1}. I, U, l, and D refer to the ionic current through the nanochannel, the voltage, the length of the nanochannel, and the diameter of the base. These results indicate the produced nanochannel is suitable for the ideal channel model.

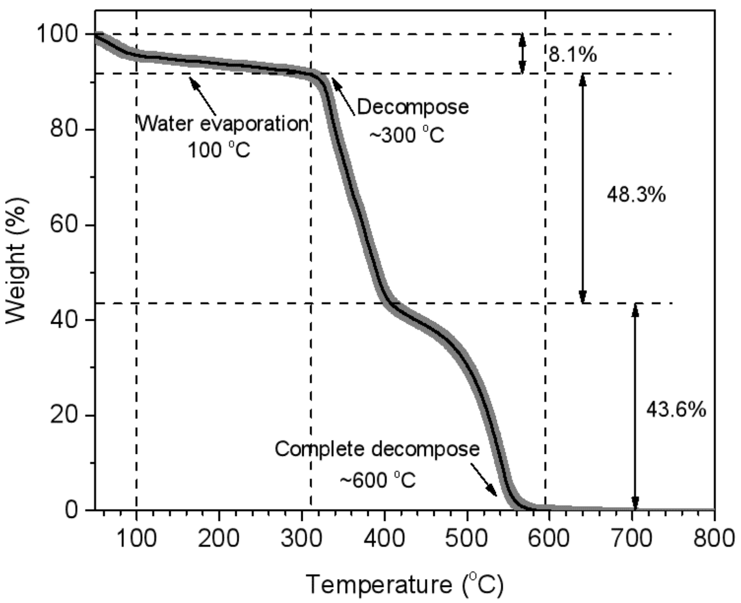

Supplementary Figure 4. TGA plot of the CPOS material. The thermogravimetric analysis (TGA) of the samples was carried out by loading the samples in an alumina pan using a SHIMADZU DTG-60 thermal analyzer at the heating rate of 10°C min^{-1} to 800°C under the dried air atmosphere with an air flow rate of 30 ml min^{-1}. TGA plots indicate that 8.1% weight loss is attributed to the water/THF removal at first 100°C. Additional 48.3% weight loss is attributed to the organic salt decomposition at ~300°C. Finally, at last 43.6% weight loss is attributed to the final degradation.

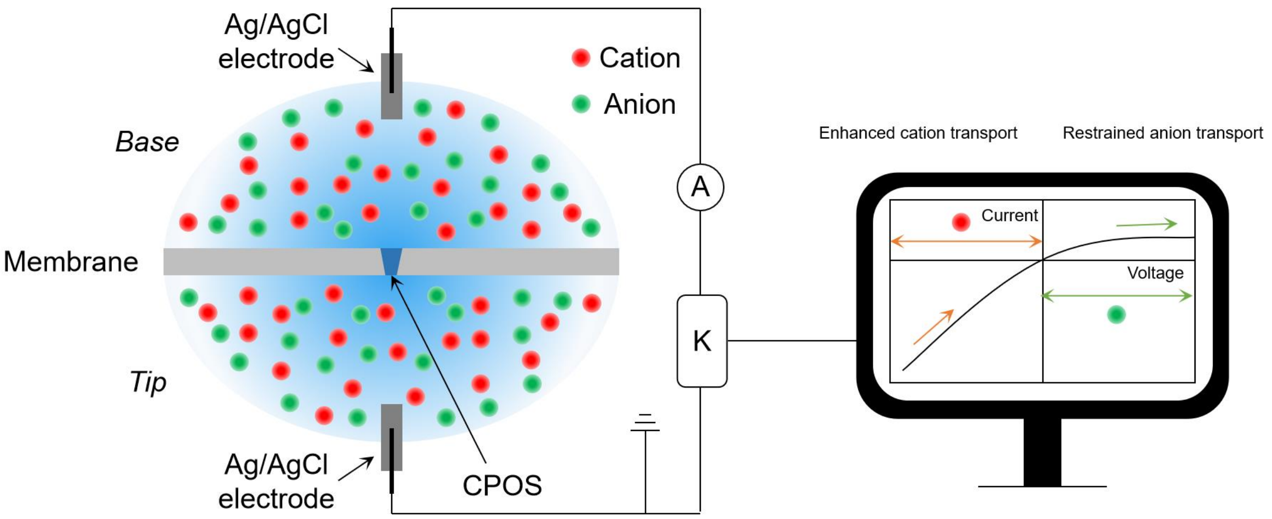

Supplementary Figure 5. The experimental set-up. The ion transport property was studied by measuring the ionic current through the biomimetic single conical nanochannel. The ionic current was measured by a picoammeter (left, K is short for Keithley Instrument; A is short for amplifier). The PI membrane with a single nanochannel in the center was mounted between two chambers of the conductivity cell with electrolytes. Homemade Ag/AgCl electrodes were used to apply a transmembrane potential across the membrane. Keithley instrument is in contact with a computer which records the *I*−*V* information (right).

A bias from −1 to +1 V with a 0.1 V step voltage was applied to gain insights into the ion transport properties across the created conically structured nanochannel. For a negatively charged conical nanochannel, only cations in electrolytes can transport through the nanochannel. At positive voltage, when cations transport, the transport behaviors become difficult due to the narrowing of the nanochannel (green arrow). On the contrary, at negative voltage, when cations transport, the transport behaviors become easy due to the broadening of the nanochannel (red arrow). It was seen that the ionic current value at +1 V is much lower than that at –1 V as shown in the right illustration.

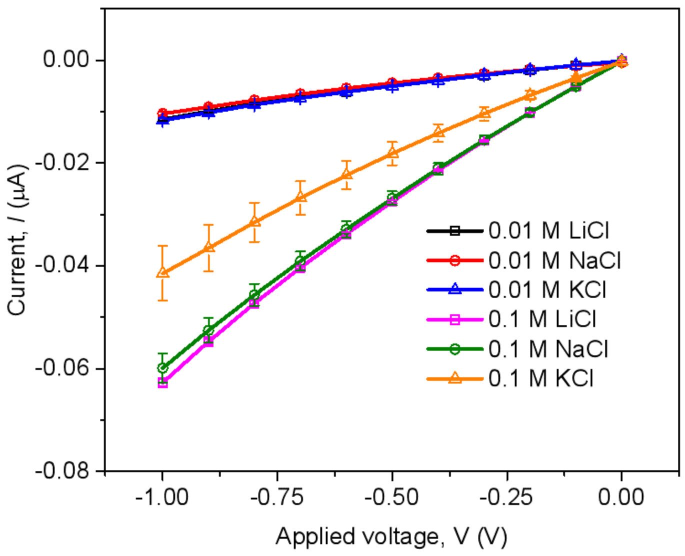

Supplementary Figure 6. The I–V characteristics of the pristine channel in 0.1 M and 0.01 M monovalent ion solutions at a bias voltage from −1 to 0 V. It was found that the channel without the CPOS show no obvious K^+ selectivity in both 0.01 and 0.1 M solutions. In particular, the ion transport behaviors in 0.01 M solutions are different from that in the CPOS, due to the larger opened size and bulk-like ion transport. In absence of the CPOS, the dimension of the channel is from 75 to 750 nm, which exceeds the thickness of the electric double layer (EDL), and thus could not show the surface-charge-governed ion transport. Error bars denote the standard deviation.

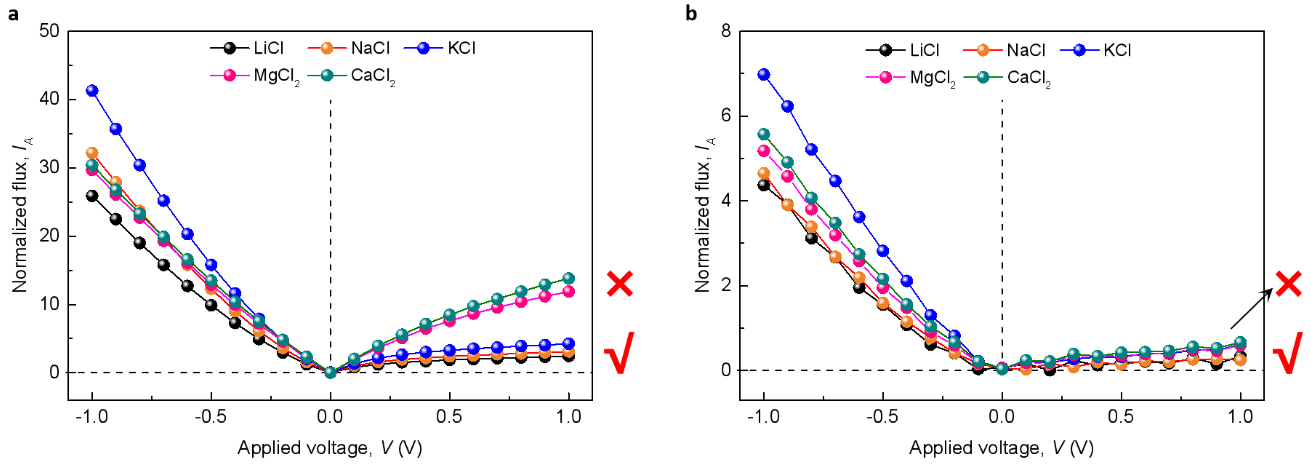

Supplementary Figure 7. The normalization flux of different salt solutions. The critical role of biomimetic K^+ channel is shown by plotting the full I_A–V relationships in which the ion flux at different applied bias were normalized to that of I_A=0. The curve profile was asymmetrically parabolic, with a sharp increase in the flux values as the bias voltage became more negative to –1.0 V, and through a single nanocone with the CPOS in 0.1 M solutions (Supplementary Fig. 7a). The normalized ion flux of $MgCl_2$ and $CaCl_2$ was higher than that of the monovalent ionic salt solutions when the applied voltage became more positive to +1.0 V, which greatly reduced the ion rectification effect. The result indicates the CPOS pores could conduct rapidly monovalent ion transport rather than bivalent ions. In addition, abundant divalent cations reversely transport from surface to bulk, which generated an ion flux at positive voltages. Monovalent cations mainly pass into the CPOS pores and thus fewer monovalent cations reversely transport. Although the weak selectivity of bivalent ions was reduced in 0.01 M solutions as shown in Supplementary Fig. 7b, the ion rectification of bivalent ions was still lower than monovalent ions. Therefore, we propose that the CPOS pores is activated in monovalent ion (K^+, Na^+, Li^+) solutions (√) instead of bivalent ion (Mg^{2+} and Ca^{2+}) solutions (×). It can be seen that the sieving of mono/bivalent ion is much easier than mono-/mono-valent ion. Note that the CPOS pores also show different monovalent ion transport behaviors in which K^+ ions exhibit the highest ionic flux.

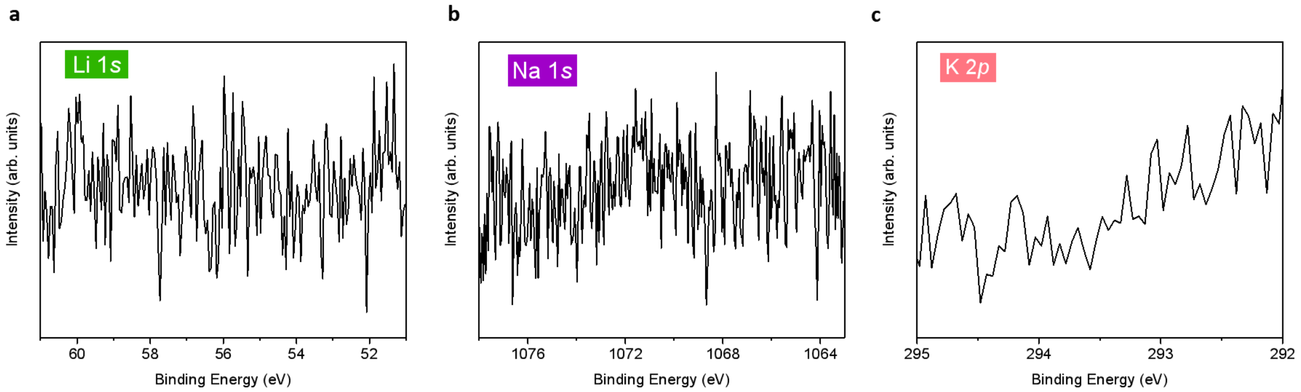

Supplementary Figure 8. High-resolution XPS spectra of the pristine CPOS samples. In the process of the *in situ* growth of the CPOS material in the channel, some salt solutions were used. In order to gain further insights into the ion distribution, the pristine CPOS samples were characterized using Li 1*s* (a), Na 1*s* (b), and K 2*p* (c) XPS spectra, respectively. Results indicate that the CPOS material were undefiled and some typical peaks were observed. In contrast to the results after ion transport, the obvious peaks of Li 1*s*, Na 1*s*, and K 2*p* situations can be found due to the binding of the CPOS and ions.

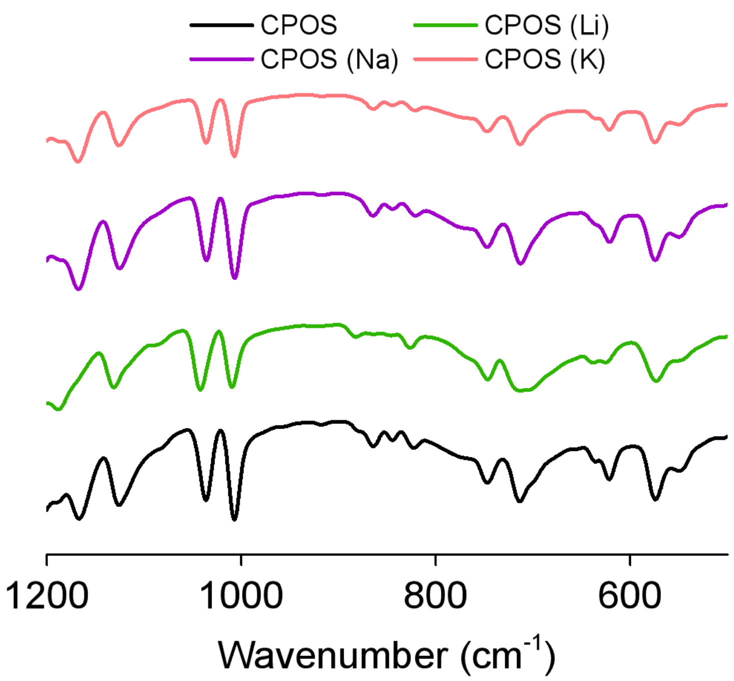

Supplementary Figure 9. FTIR spectra of the CPOS material at low wavenumber. 1169(m), 1128(m), 1080(w), 1038(m), 1009(s), 880(w), 864(w), 845(w), 826(w), 743(m), 716(m), 637(m), 623(m), 573(w), 465(w).

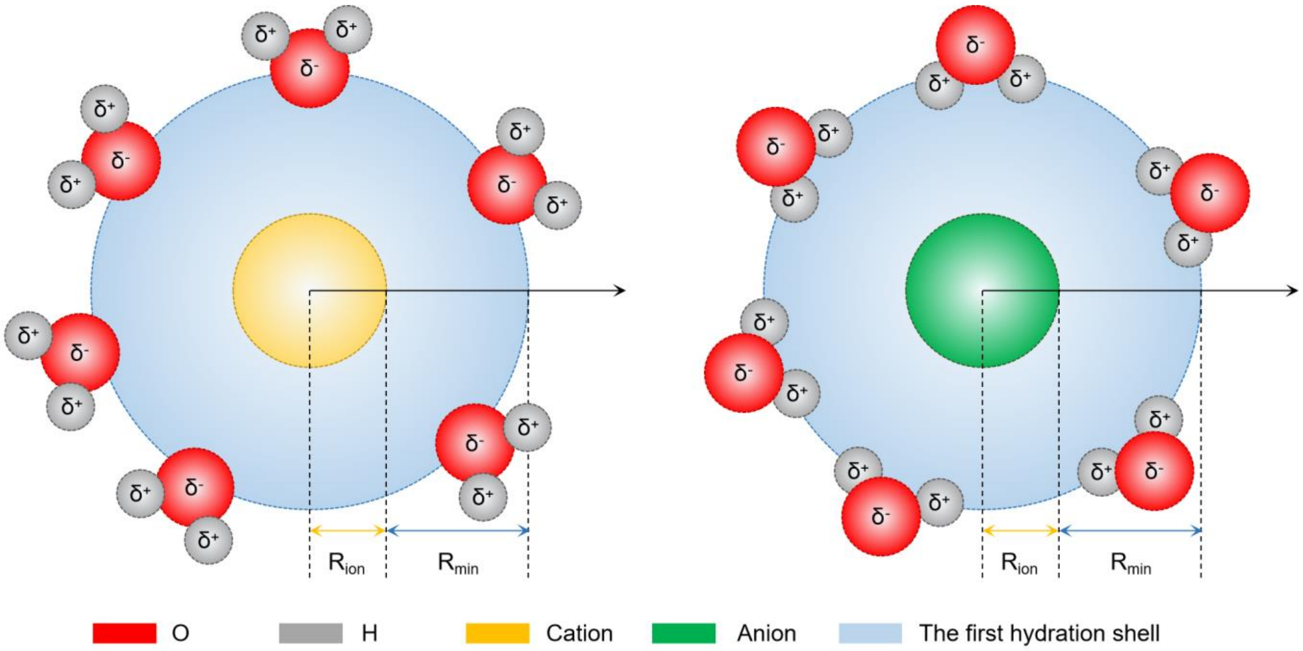

Supplementary Figure 10. Schematic of ion radius (R_{ion}) and the first hydration shell radius (R_{min}). The first hydration shell could produce an obvious interaction with the CPOS, playing a role in ion transport. When ions transfer in nanoconfinement, hydration ions could lose the binding water molecules, especially the first hydration shell. As shown in the right schematic, the cation is in contact with the aspect of the oxygen (δ^{-}). In contrast, anion (Cl^{-}) is in contact with the aspect of the hydrogen (δ^{+}). Detailed radius of the first hydration shell can be found in Supplementary Table 1.

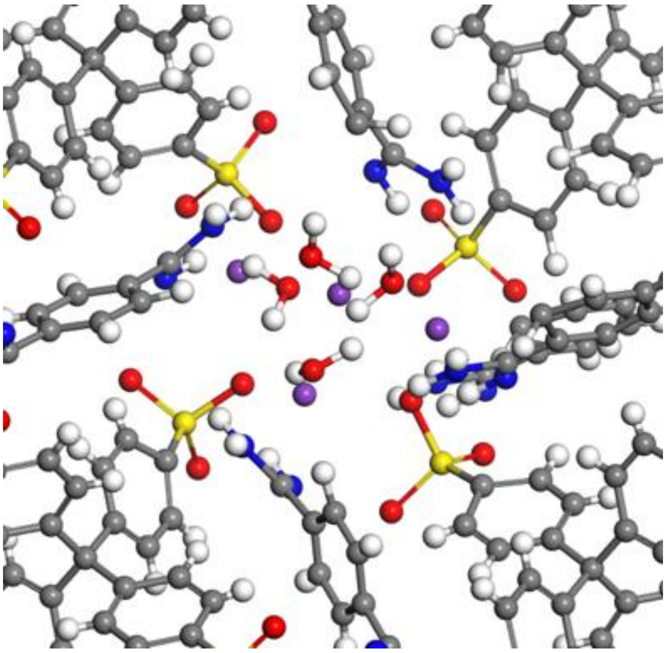

Supplementary Figure 11. The molecular snapshot of ions in the center of the screwing cavity. The simulation was set in water environments to obtain further information on the ion transport.

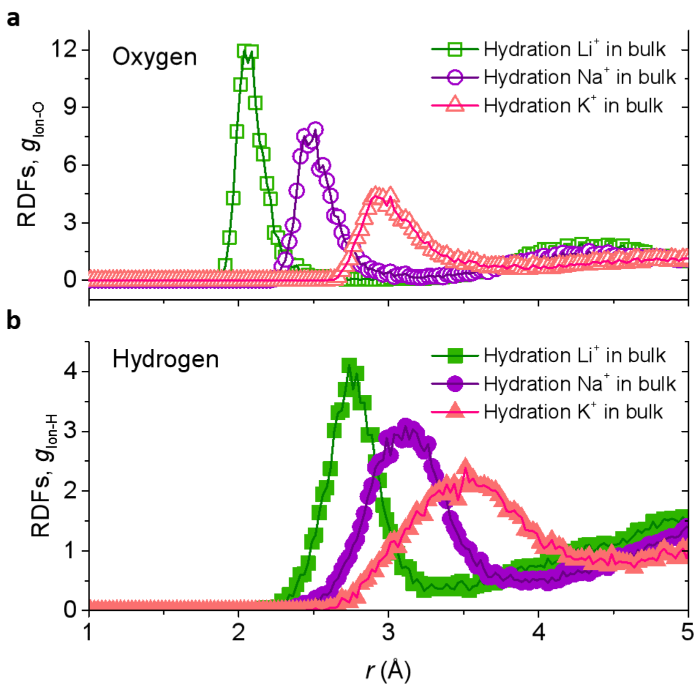

Supplementary Figure 12. RDFs of water molecules around anions sitting in bulk water. RDFs of the ion–oxygen (a) and ion–hydrogen (b) distances in bulk water calculated using the same force-field with that in CPOS pores. It can be found that the distance of oxygen from ion in bulk water (a) becomes longer than that in CPOS pores (Fig. 5a) due to the interaction between oxygen atoms and the walls of CPOS pores. Similarly, the hydrogen atoms are more closed to the metal ion due to the rotation of hydrated water molecular in nanoconfined CPOS pores (b). These calculated data are consistent with the experimental results.

a

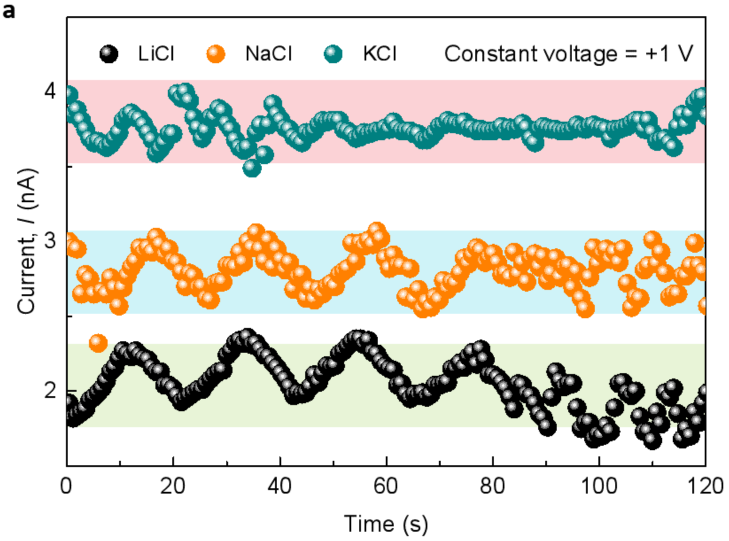

b

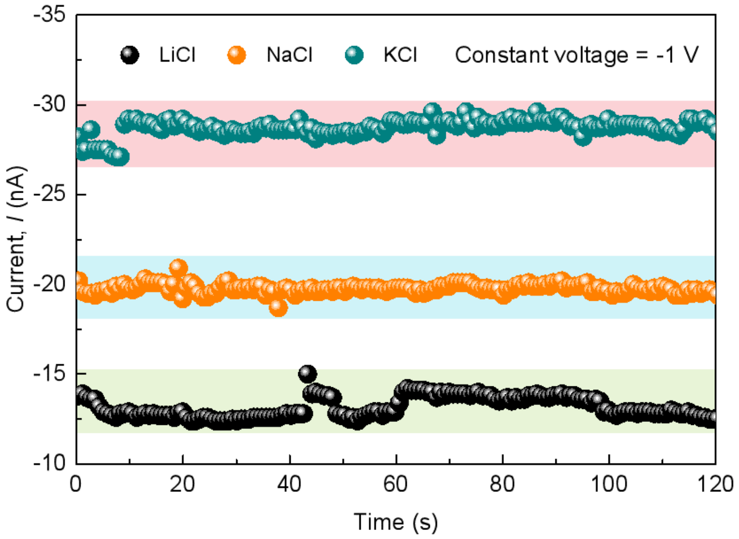

Supplementary Figure 13. Ionic current under a constant bias. For comparison, two constant biases of +1 V (a) and −1 V (b) were respectively applied to confirm the effective voltage control for the high-performance ion selectivity. The ionic current at +1 V is one order of magnitude lower than that at −1V, which is in good agreement with the results in Supplementary Fig. 5. In addition, the selectivity at −1V is higher than that at +1 V. Therefore, the bias of −1 V was used in next experiments. The biomimetic K^+ channel membrane was clamped between two PTFE compartments, of which one cell, facing the base of the conically structured nanochannel, was filled with a ternary ion mixture containing LiCl, NaCl, and KCl with the same concentration as the feed solutions, while the other cell was filled with MilliQ water as the permeation solution. The ternary ion selectivity and transport property

measurements were conducted by applying a constant potential of −1 V via a Keithley 6487 for 24 h using Pt electrodes. At the end of these experiments, the ion concentrations in the permeation side were measured using the inductively coupled plasma mass spectrometry.

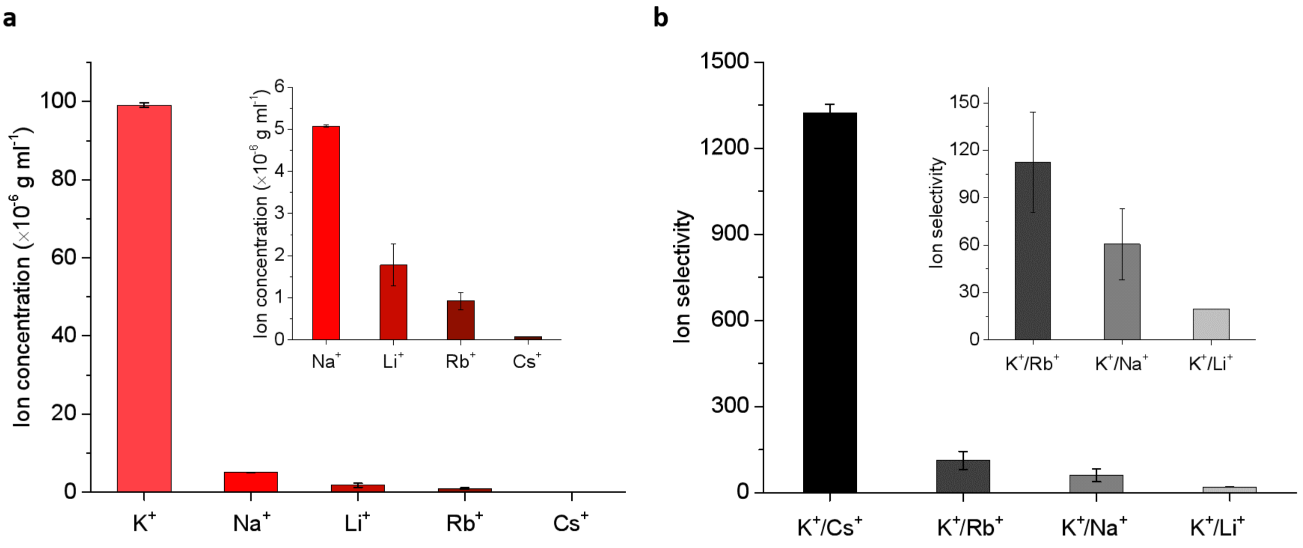

Supplementary Figure 14. Ion selectivity in the quinary solutions. (a) Ion permeation through the CPOS pores under an external bias of −1 V. (b) Ion selectivity of the CPOS pores. We performed the ion permeation experiments to show the selectivity of Cs^+ and Rb^+ in a mixture (100 mM LiCl, NaCl, KCl, RbCl, and CsCl as the feed solutions) through the CPOS pores. The other cell was filled with MilliQ water as the permeation solution. The ion concentration in the permeation was obtained by the inductively coupled plasma mass spectrometry (ICP-MS) measurements. As shown in Supplementary Fig. 14a, the CPOS pores have a selective permeation with a rank of $K^+ >> Na^+ > Li^+ > Rb^+ > Cs^+$, which show the ultra-selective K^+ transport. According these results, the permeation of Rb^+ is larger than that of Cs^+ because Rb^+ has a smaller ion radius and radius of the first hydration shell (see Supplementary Table 1). In this case, the Rb^+ could more easily enter and pass the CPOS pores. Although the hydration energy of Cs^+ is lower than that of Rb^+, the size effect might play a more important role in the selective transport. Based on the permeation results, the ion selectivity of K^+/Cs^+ and K^+/Rb^+ is up to ~1300 and ~110, which is an excellent advance in the monovalent ion sieving (Supplementary Fig. 14b). Additionally, the ion selectivity of K^+/Li^+ and K^+/Na^+ drops due to the competitive penetration in quinary mixed solutions. These results reveal that this CPOS materials could show potential in the removal of the radioactive element such as isotopes of cesium. Error bars denote the standard deviation.

3. Supplementary Tables

Supplementary Table 1. Ion-dependent parameters including the ionic radius, hydration ionic radius, number of the first hydration shell, and hydration energy.

Ion type	Ionic radius[4] (Å)	Hydration ionic radius[4] (Å)	Radius (R_{min}) of the first hydration shell[5] (Å)	Hydration energy[5] (kcal mol^{-1}) in kT
Li^+	0.60	3.82	2.08	122.2±0.6
Na^+	0.95	3.58	2.36	98.8±0.8
K^+	1.33	3.31	2.80	80.9±1.0
Rb^+	1.48	3.29	2.89	75.5±0.9
Cs^+	1.69	3.29	3.14	67.7±0.7
Mg^{2+}	0.65	4.28	2.09	455.2±0.3
Ca^{2+}	0.99	4.12	2.42	380.8
Cl^-	1.81	3.32	2.24	75.8

The size of the first hydration shell of K^+ ions is larger than those of Mg^{2+} and Ca^{2+} ions (Supplementary Table 1), while the ionic flux and selectivity are consistent with the lower hydration energy of K^+ ions.

Supplementary Table 2. Monovalent ion selectivity based on various types of the artificial channels and membranes. Typical parameters including the selectivity of K^+/Li^+, K^+/Na^+ and Na^+/Li^+, ion conductivity, ion transport rate, and applied bias, were listed in the table.

Materials	K^+/Li^+	K^+/Na^+	Na^+/Li^+	Ion conductivity ($S\ m^{-1}$)	Transport rate ($mol\ m^{-2}\ h^{-1}$)	Driving force	Feed solution concentration (M)	Reference
PET lumirror film	1.5	1.19	1.26	0.19	13.9	+10 V	1.0	6
PET Hosaphan film	0.65	0.062	0.096	2.61×10^{-4}	2.3×10^{-3}	+10 V	1.0	7
PET nanopore	1.50	1.56	–	–	–	+2 V	0.1	8
MoS_2 nanopore	0.76	0.43	1.76	17.8	–	+0.8	1.0	Fig. 5f
GO membrane	–	1.1	–	–	3.0	ΔC	1.0	Fig. 5f
PSS/HKUST−1 membrane	0.015	0.52	0.029	5.6×10^{-4}	0.1	0.4 V<br>ΔC	0.5	9
Zwitterion−carbon nanotube membrane	–	0.98	–	–	–	ΔP	–	10
Asy-MOFSNC	2.6	1.2	2.2	1.95	–	+1 V	0.1	Fig. 5f
Lyotropic liquid crystal membrane	–	1.44	–	–	1.3×10^{-4}	ΔP	–	Fig. 5f

Graphene nanopores with carboxylate groups	–	~2	–	–	–	+0.654V	0.5	Fig. 5f
i–GO membrane	1.04	1.08	0.96	–	1.36	ΔC	0.5	11
Al_{13}–$Ti_3C_2T_x$ lamellar membrane	1.2	1.0	1.2	–	0.01	ΔC	0.5	12
Isolated sub-2-nm graphene nanopores	2	–	–	–	–	0.2 V	0.1	Fig. 5f
Gr/MoS_2 2D channel	1.35	1.15	1.18	1.2	–	+0.2 V	0.1	13
Graphene nanopore	1.48	1.18	1.26	1.14	–	+0.15 V	0.1	Fig. 5f
rGO membrane	14.08	3.52	4	–	0.19	ΔC	0.1	Fig. 5f
Crosslinked GO	–	1.5	–	–	–	ΔC	0.1	Fig. 5f
Polysulfone Gr	–	2.6	–	–	0.6	ΔC	0.5	Fig. 5f
Physically confined GO	1.3	1.55	0.84	–	0.007	ΔC	1.0	14
Nitrogen–doped Gr	–	4.875	–	–	–	ΔC	0.1	Fig. 5f
Sulfonated MOFs	1.43	1.14	1.25	–	–	+1 V<br>ΔC	1.0	15
Potassium activated	~5	3.33	1.5	–	–	2 V	0.01	16

nanochannels								
Cation-controlled GO membrane	–	1.15	–	–	0.23	ΔC	0.25	Fig. 5f
Biomimetic potassium-selective nanopores	–	78	–	–	–	1 V	1.0	Fig. 5f
SWCNT network membrane	–	1.2	–	–	$< 6 \times 10^{-3}$	ΔC	0.1	17
Electrochemically tunable ion-sieve membrane	5.8	4.8	1.2	–	6.9×10^{-2}	ΔC	0.1	18
The smallest graphene flakes (sGP) membrane	2	1.6	1.25	–	–	ΔC	0.1	19
$Ti_3C_2T_x$ membrane	0.67	0.61	1.09	–	0.94	ΔC	0.2	20
α-Al_2O_3 plate membrane	–	7.62	–	–	0.59	+6 V	–	21
Atomic-scale graphene channels	9	–	–	–	~4.6	−1 V	0.2	Fig. 5f
Biomimetic K^+ channels	363.8	31.6	11.5	73.1	9.44×10^{-2}	−1 V<br>ΔC	0.1	This work

Supplementary Table 3. Experimental details of the zero-current voltage (E_m), E_{redox}, and real E_m values which could be calculated according the equation: real E_m=E_m-E_{redox}.

Entry	γ^a	γ^b	E_m (mV)	E_{redox} (mV)	real E_m (mV)
LiCl	0.756	0.900	66.1	54.6	11.5
NaCl	0.780	0.904	76.4	55.3	21.1
KCl	0.790	0.901	95.9	55.8	40.1

[a]Activity coefficient in 0.1 M solutions.

[b]Activity coefficient in 0.01 M solutions.

4. Supplementary References

1 Sarma B., Nangia A. Tetrakis(4-sulfophenyl)methane dodecahydrate. Reversible and selective water inclusion and release in an organic host. *CrystEngComm* 9, 628-631 (2007).

2 Liu Q., Xiao K., Wen L., Lu H., Liu Y., Kong X. Y., Xie G., Zhang Z., Bo Z., Jiang L. Engineered ionic gates for ion coduction based on sodium and potassium activated nanochannels. *J. Am. Chem. Soc*. 137, 11976-11983 (2015).

3 Siwy Z. S. Ion-current rectification in nanopores and nanotubes with broken symmetry. Adv. Funct. Mater. 16, 735-746 (2006).

4 Nightingale, E.R. Phenomenological theory of ion solvation. Effective radii of hydrated ions. *J. Phys. Chem*. 63, 1381-1387 (1959).

5 Marcus, Y. A simple empirical-model describing the thermodynamics of hydration of ions of widely varying charges, sizes, and shapes. *Biophys. Chem*. 51, 111-127 (1994).

6 Wang P., Wang M., Liu F., Ding S., Wang X., Du G., et al. Ultrafast ion sieving using nanoporous polymeric membranes. *Nat. Commun*. 9, 569 (2018).

7 Wen, Q., Yan, D.X., Liu, F., Wang, M., Ling, Y., Wang, P.F., et al. Highly selective ionic transport through subnanometer pores in polymer films. *Adv. Funct. Mater*. 26, 5796-5803 (2016).

8 Gamble T., Decker K., Plett T. S., Pevarnik M., Pietschmann J. F., Vlassiouk I., Aksimentiev A., Siwy Z. S., Rectification of ion current in nanopores depends on the type of monovalent cations: experiments and modeling. *J. Phys. Chem. C* 118, 9809-9819 (2014).

9 Guo, Y., Ying, Y.L., Mao, Y.Y., Peng, X.S. & Chen, B.L. Polystyrene sulfonate threaded through a metal-organic framework membrane for fast and selective lithium-ion separation. *Angew. Chem. Int. Ed*. 55, 15120-15124 (2016).

10 Liu, T.Y., Yuan, H.G., Li, Q., Tang, Y.H., Zhang, Q., Qian, W.Z., et al. Ion-responsive channels of zwitterion-carbon nanotube membrane for rapid water permeation and ultrahigh mono-/multivalent ion selectivity. *ACS Nano* 9, 7488-7496 (2015).

11 Zhang M., Zhao P., Li P., Ji Y., Liu G., Jin W., Designing biomimic two-dimensional ionic transport channels for efficient ion sieving. *ACS Nano* 15, 5209-5220 (2021).

12 Zhu J., Wang L., Wang J., Wang F., Tian M., Zheng S., Shao N., Wang L., He M., Precisely tunable ion sieving with an Al_{13}-$Ti_3C_2T_x$ lamellar membrane by controlling interlayer spacing. *ACS Nano*

14, 15306-15316 (2020).

13 Esfandiar A., Radha B., Wang F.C., Yang Q., Hu S., Garaj S. et al. Size effect in ion transport through angstrom-scale slits. *Science* 358, 511-513 (2017).

14 Abraham J., Vasu K.S., Williams C.D., Gopinadhan K., Su Y., Cherian C.T. et al. Tunable sieving of ions using graphene oxide membranes. *Nat. Nanotechnol*. 12, 546-550 (2017).

15 Li X., Zhang H., Hou J., Ou R., Zhu Y., Zhao C., Qian T., Easton C. D., Selomulya C., Hill M. R., Wang H., Sulfonated sub-1-nm metal-organic framework channels with ultrahigh proton selectivity. *J. Am. Chem. Soc*. 142, 9827-9833 (2020).

16 Liu Q., Xiao K., Wen L., Lu H., Liu Y., Kong X. Y., Xie G., Zhang Z., Bo Z., Jiang L., Engineered ionic gates for ion conduction based on sodium and potassium activated nanochannels. *J. Am. Chem. Soc*. 137, 11976-11983 (2015).

17 Gao S., Zhu Y., Gong Y., Wang Z., Fang W., Jin J., Ultrathin polyamide nanofiltration membrane fabricated on brush-painted single-walled carbon nanotube network support for ion sieving. *ACS Nano* 13, 5278-5290 (2019).

18 Li Z. Q., Wu M. Y., Ding X. L., Wu Z. Q., Xia X. H., Reversible electrochemical tuning of ion sieving in coordination polymers. *Anal. Chem*. 92, 9172-9178 (2020).

19 Hirunpinyopas W., Iamprasertkun P., Bissett M. A., Dryfe R. A. W., Tunable charge/size selective ion sieving with ultrahigh water permeance through laminar graphene membranes. *Carbon* 156, 119-129 (2020).

20 Ren C. E., Hatzell K. B., Alhabeb M., Ling Z., Mahmoud K. A., Gogotsi Y., Charge- and size-selective ion sieving through $Ti_3C_2T_x$ MXene membranes. *J. Phys. Chem. Lett.* 6, 4026-4031 (2015).

21 An S.-S., Liu J., Wang J.-H., Wang M.-C., Ji Z.-Y., Qi S.-S., Yuan J.-S., Synthesis and characterization of a plat sheet potassium ion sieve membrane and its performances for separation potassium. *Sep. Purif. Technol.* 212, 834-842 (2019).
